# Modeling and Parametrization of Size-Dependent Processes in Multiphase Aerosol Chemistry

**DOI:** 10.1021/acsestair.5c00486

**Published:** 2026-06-04

**Authors:** Sandhya Sethuraman, Daniel M. Westervelt, Kedong Gong, Vicki H. Grassian, V. Faye McNeill

**Affiliations:** † Department of Chemical Engineering, 5798 Columbia University, New York, New York 10027, United States; ‡ Lamont-Doherty Earth Observatory, 5798 Columbia University, Palisades, New York 10964, United States; § Climate School, 5798 Columbia University, New York, New York 10027, United States; ∥ Department of Chemistry, 8784 University of California San Diego, La Jolla, California 92093, United States; ⊥ Department of Earth and Environmental Sciences, 5798 Columbia University, New York, New York 10027, United States

**Keywords:** aerosol, kinetics, mass transfer, microdroplet, interface, atmospheric chemistry

## Abstract

Many atmospherically relevant multiphase reactive systems exhibit size-dependent kinetics in laboratory studies, with apparent reaction rates increasing with decreasing droplet size, suggesting an important role for interfacial processes. Here, we present CHAI (CHemistry of Aerosol Interfaces), a physicochemical modeling framework that describes these reactive systems using an additive resistance approach, considering the various mass transfer and reaction processes taking place simultaneously in the gas phase, droplet bulk, and at the droplet surface, and their relative time scales. We demonstrate its applicability to modeling and developing parametrizations for several inorganic and organic oxidation processes in microdroplets and aerosols, including S­(IV) to S­(VI) conversion, through simulation of experimental data. CHAI is also used to reconcile single-droplet observations with chamber and flow tube experiments and is extended to parametrization of these processes for representation in large-scale atmospheric models. Our results show that neglecting the size dependence of aerosol reaction processes can lead to inaccuracies in atmospheric chemistry modeling, motivating the CHAI approach for modeling these multiphase reactive systems and further experimental studies.

## Graphical abstract

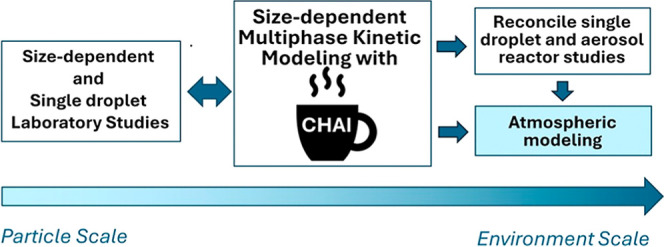

## 1 Introduction

Multiphase chemical processes (i.e., gas–droplet reactions) impact stratospheric and tropospheric chemistry, secondary inorganic and organic aerosol formation, aerosol–climate interactions, and human health.^1^ Typically, multiphase processes are complex and involve several coupled mass transfer and reaction steps, including the partitioning of the gas-phase reactant into the condensed phase, adsorption and desorption, solvation and desolvation, diffusion of gas and liquid phase species, and chemical reactions at the interface and in the bulk liquid.^2^ Determining the relative importance of each process generally requires a comparison of their characteristic time scales. There is compelling evidence suggesting that several reactions that proceed slowly in bulk solutions are accelerated at the surface of small droplets, with acceleration factors of up to 10^6^,^3,4^ although the reverse trend has also been observed.^5^ The observed rate may be accelerated either due to a concentration effect (i.e., higher density of reactant at the interface than in the bulk) or the acceleration of the reaction itself at the surface. A variety of explanations for the acceleration of reactions at the interface have been proposed, including the orientation and partial solvation of reactants,^6^ pH gradients (superacidity) at the gas–liquid surface,^7^ or interfacial electric field effects in particles.^8–10^ Other possible effects, including ionic strength effects in droplets, charged droplets, and evaporation phenomena, could explain experimental trends.^11^

Sulfate is a major component of fine particulate matter (PM_2.5_), contributing to local and regional haze formation and acid rain. It is formed through the gas-phase atmospheric oxidation of reduced sulfur (S­(IV)) compounds, particularly SO_2_. While SO_2_ oxidation to form H_2_SO_4_ does take place, the majority of particulate matter sulfate is believed to be formed through multiphase processes, especially oxidation by O_3_ and H_2_O_2_ in cloudwater.^12^ Although S­(IV) oxidation in aerosol water was first proposed in 1982 by Hering and Friedlander,^13^ this process does not dominate sulfate production in North America and Europe, and has historically not been prioritized for air quality modeling. However, it has received increased attention in the past decade due to its likely role in haze formation in urban China.^14^ Due to the sensitivity of the primary S­(IV) oxidation pathways to pH and ionic strength,^15^ extrapolation of kinetic parameters from dilute cloudwater conditions to aerosol conditions is not possible, so there have been a large number of laboratory studies in recent years aimed at characterizing these processes in aerosols. Several single-droplet kinetics studies of S­(IV) → S­(VI) oxidation have inferred a special role for interfacial processes based on observations that the apparent reaction rate varies with droplet size, and therefore surface-to-volume ratio.^16–18^ Generally, faster processing rates are observed in smaller particles, suggesting that interfacial processing is faster than bulk reactions. Observations extended to large droplet sizes demonstrate a size beyond which bulk chemistry dominates over interfacial processes.^18^ This critical droplet size is typically much larger than atmospherically relevant aerosol sizes, suggesting that surface processes and size-dependent chemistry are significant for modeling aerosol-phase S­(IV) oxidation in the atmosphere.

Given the possible importance of fast interfacial processes to atmospheric phenomena such as haze formation, a modeling approach is needed to simulate these processes, and provide a pathway for their representation in large-scale models. A framework is also needed to reconcile the results of single-droplet laboratory studies and aerosol flow tube or aerosol chamber studies, to ensure consistent interpretation of these results across different size regimes as well as different experimental methods. Following fundamental treatments of mass transfer and reaction in gas–liquid systems by Danckwerts,^19–21^ several approaches have been proposed for modeling the interactions between liquid droplets or aerosols and gas-phase oxidants. Schwartz and co-workers developed a framework of differential equations for modeling the dynamics of mass transfer and in-droplet reaction for cloud processes.^22^ Hanson, Ravishankara, and Solomon developed an analytical framework for reactions in liquid sulfuric acid aerosols for stratospheric chemistry.^23^ Worsnop and co-workers explored limiting cases of multiphase atmospheric chemistry (e.g., uptake controlled by fast or slow bulk reaction, gas phase or particle phase diffusion, or a surface process) using a resistance equation, wherein the overall reactive uptake coefficient (γ) is expressed as the sum of contributions from several decoupled processes.^2^ Multilayer models have been an alternative approach to simulating reaction and mass transport in coupled microcompartments including the gas–particle interface, and have successfully been used to simulate multiphase systems, including transient dynamics and transport limitations.^24–28^ In a more recent effort to develop a model that is valid for systems that fall outside the canonical limiting cases, Wilson et al. developed a physically constrained stochastic kinetic model to predict the time scales of reactions in levitated droplets, dividing droplets up into microcompartments (including a surface and bulk region), and constraining each known elementary reaction, partitioning, or diffusion step with rate coefficients prior to simulation.^29–31^

Recently, we applied an additive resistance approach to model single-droplet experimental observations of the size-dependent uncatalyzed oxidation of SO_3_^2–^ in microdroplets over a large size range.^16^ We showed that the observed kinetics, including the size dependence, was represented well by a simple two-parameter model representing a slow bulk reaction in series with a relatively efficient surface process. In this study, we extend this this approach, which we term CHAI (CHemistry of Aerosol Interfaces), to model and derive parametrizations for several other reactive systems which show aerosol size-dependent kinetics, spanning a range of relative reaction and mass transport time scales.

In addition to using CHAI to study single droplet systems, we use CHAI to reconcile the kinetics in single droplet experiments and in a polydisperse aerosol population (i.e., in an atmospheric chamber or flow tube), and show that neglecting the size dependence of these processes can lead to significant inaccuracies in modeling for particles in the atmospherically relevant submicron size range. We further discuss differences in kinetic trends from bulk solution phase chemistry studies compared to small aerosols and droplets. We also provide a pathway for using CHAI-derived parametrizations to represent these complex multiphase reactions in large-scale atmospheric models.

## 2 Methods and Model Description

In multiphase systems, several processes, including gas or in-particle diffusion, mass accommodation, and reactions in the bulk or at the surface of the particle, could be taking place in series or in parallel. These processes all contribute to the observed kinetics. The relative time scales of these processes determine the overall rate of the process, the characteristic shape of the species concentration profiles, and its dependence on particle size, reactant concentration, and temperature. In an additive resistance formulation,^2^ these processes contribute to the observed reactive uptake coefficient, γ_*obs*_, as follows

1 $$\frac{1}{\gamma_{obs}}=\frac{1}{\gamma_{diff}}+\frac{1}{\gamma_{p,diff}}+\frac{1}{S}+\frac{1}{\gamma_{s}{+\gamma }_{rxn}}$$

where 1/γ_diff_ is the resistance due to gas-phase diffusion of reactants to the surface, 1/γ_p,diff_ is the particle-phase diffusion resistance, and 1/*S* is the resistance due to adsorption at the interface. γ_s_ and γ_rxn_ represent surface uptake and reaction within the particle, respectively. The functional forms of each of these terms are provided in the Supporting Information (eq S1–S5). There are several typical limiting cases under which eq 1 may be simplified, which were outlined by Worsnop and coauthors.^2^ These include either a fast reaction, with comparatively slower in-particle diffusion, or a slower particle phase reaction, with comparatively faster in-particle diffusion. These cases, common for heterogeneous chemistry of organic aerosols, are not always applicable for a reactive systems exhibiting size-dependent kinetics and moderate reaction rates, such as uncatalyzed oxidation of S­(IV) by O_2_ to form S­(VI) (Figure 1),^16,18^ but may be applicable to systems such as S­(IV) oxidation by NO_2_ or O_3_, which exhibit faster bulk reaction rates (Table S1).^32–35^

**Figure 1:**
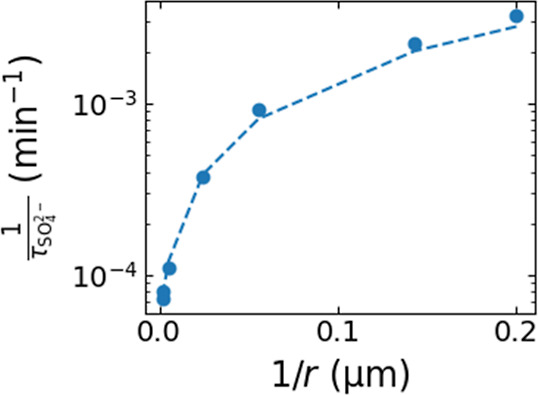
Sulfate formation time scale vs radius for the oxidation of S­(IV) with O_2_ at pH 9. Calculated using experimental data from Li et al.^18^ See text for details.

To illustrate this point, we calculate the time scales for bulk reaction (in the absence of mass transfer limitations) and in-particle diffusion as follows

2 $$\tau_{bulk}=\frac{1}{HRTn_{x\left(g\right)}k_{2}}$$

3 $$\tau_{p,diff}=\frac{a^{2}}{D}$$

In these equations, *a* is the radius of the particle (m), R is the universal gas constant (L atm mol^–1^ s^–1^), *T* is the temperature (K), *H* is the Henry’s Law coefficient (M atm^–1^), *n*_*x*(g)_ is the gas phase concentration (M), *k*_2_ is the bimolecular bulk rate constant (M^–1^ s^–1^), and *D* (m^2^ s^–1^) is the in-particle diffusivity of an aqueous-phase species (either S­(IV) or the oxidant).

In Figure 2, we compare the time scales of diffusion for S­(IV) and the oxidant, the time scale for bulk reaction estimated using *k*_2_ values previously reported in the literature (Table S1) for aerosol systems^32,33^ where possible or else bulk solutions,^34–37^ and the experimentally observed time scales for S­(IV) oxidation reported by Gong et al.^16^ and Li et al.^18^ as a function of particle size.

**Figure 2:**
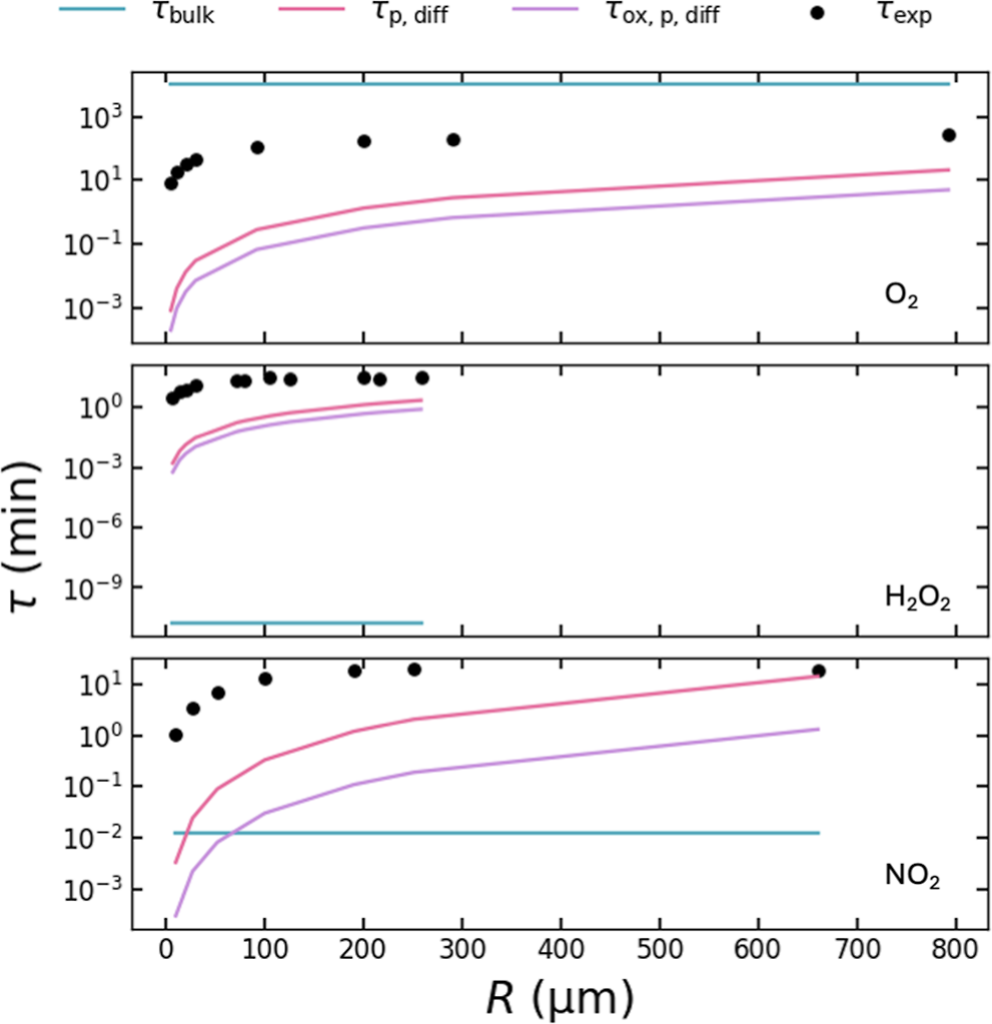
Time scale analysis for S­(IV) → S­(VI) oxidation by O_2_, H_2_O_2_, and NO_2_ at pH ∼ 5, compared with time scales of S­(IV) oxidation experimentally reported by Gong et al.^16^ and Li et al.^18^ See text for details.

For cases where the bulk reaction is sufficiently slow, such as uncatalyzed O_2_ oxidation, time scales of in-particle S­(IV) and oxidant diffusion (τ_p,diff_ and τ_p,ox,diff_) are substantially (∼2 orders of magnitude or more) faster than the bulk reaction and experimentally observed time scales, confirming a reaction-limited regime. The bulk reaction time scale is much slower than the experimentally observed oxidation, suggesting a role for an efficient interfacial process.^16^ By contrast, for both H_2_O_2_ and NO_2_ oxidation at pH 5, the bulk rates are faster than experimentally observed, and faster than in-particle diffusion for most particle sizes, suggesting a mass-transport limited regime where oxidant concentrations may be nonuniform within the particle.

To further examine the issue of transport limitations, we plot τ_bulk_ and τ_p,ox,diff_ using *k*_2_ values from Table S1 and the experimental conditions of Li et al. ([*Y*] = 2.6 M and *a* = 20 μm) (Figure 3).

**Figure 3:**
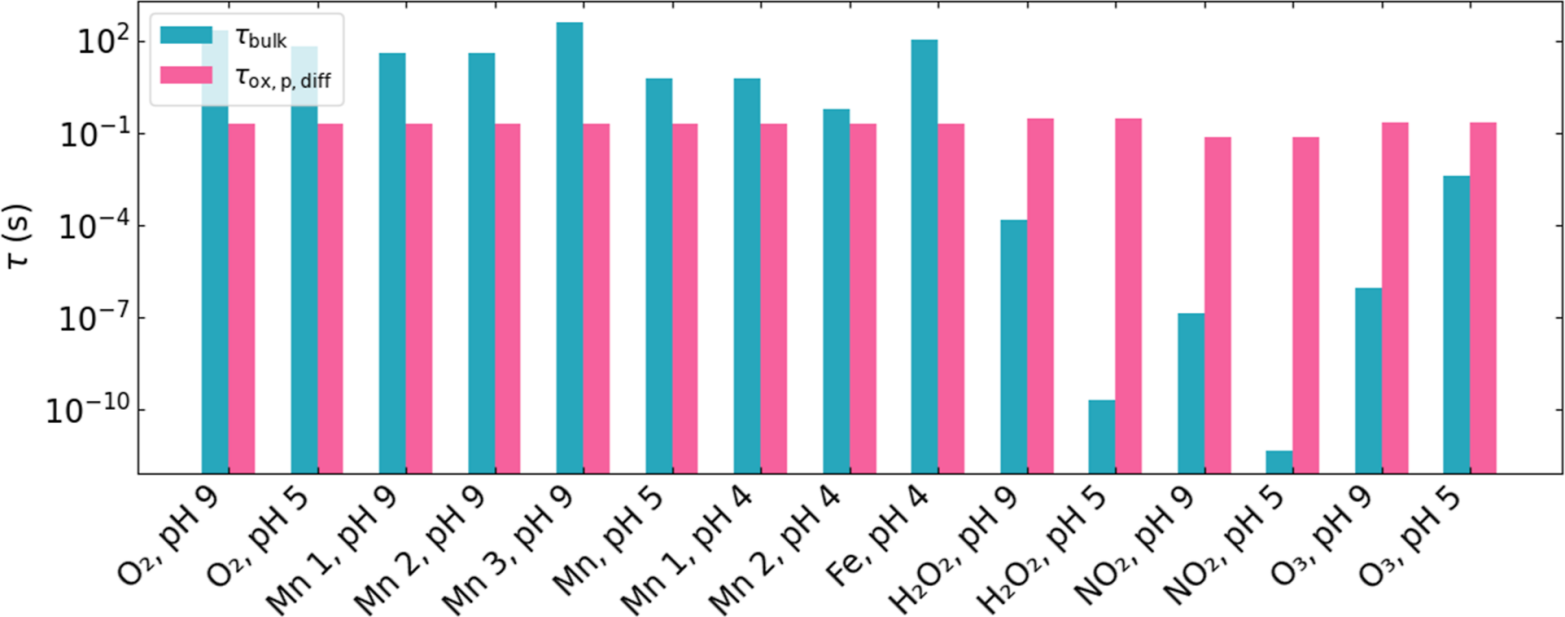
Bar charts comparing τ_bulk_ (1/*k*_2_[*Y*]) from literature to τ_p,ox,dif*f*_ (1/*k*_transport_) following Table S1.

In line with the time scale analysis above, τ_bulk_ > τ_p,ox,diff_ in the case of uncatalyzed and catalyzed O_2_ oxidation. For the other oxidant systems (H_2_O_2_, NO_2_, and O_3_), τ_bulk_ < τ_*p*,*ox*,*diff*_, consistent with a transport limited regime. For several systems, including Mn­(II) catalyzed oxidation at low pH and O_3_ oxidation at pH 5, τ_bulk_ ∼ τ_p,ox,diff_, suggesting reaction and transport are both important. We note that direct application of rate constants measured in dilute bulk solutions to the aerosol “bulk” is not always appropriate due to ionic strength effects, salting in/out effects, and the highly acidic droplet environment. Therefore, as we derive *k*_2_ for aerosol and droplet systems we revisit the possibility of transport limitation in an iterative process.

In the reaction-limited case, CHAI represents the overall uptake coefficient by the resistances of the surface reaction and bulk reaction in series.

4 $$\gamma_{obs}=\gamma_{s}+\gamma_{rxn}$$

5 $$\gamma_{s}=\frac{4RT\beta \left[Y\right]}{w}$$

6 $$\gamma_{rxn}=\frac{4aHRT}{3w}k_{2}\left[Y\right]$$

In this formulation, [*Y*] is the concentration (M) of the aqueous phase reactant within the droplet, and *w* is the mean thermal velocity

7 $$w=\sqrt{\frac{8k_{B}T}{\pi m}}$$

where *k*_*B*_ is the Boltzmann constant and *m* is the mass of a single gas molecule (kg). β is a compound parameter that represents surface uptake and reaction

8 $${\beta =k}_{2,s}H_{s}K_{s}$$

Here, *k*_*2*,*s*_ and *H*_*s*_ are the surface analogs of the Henry’s Law coefficient and the rate constant, and *K*_*s*_ represents the equilibrium constant for the partitioning of S­(IV) to the surface.

When the system is transport-limited, CHAI represents the overall uptake coefficient using a single parameter (*k*_2_).

9 $$\gamma_{rxn}=\frac{4HRT}{w}\sqrt{D_{x\left(p\right)}k_{2}\left[Y\right]}$$

where *D*_*x*(*p*)_ is the in-particle diffusivity of the gas-phase oxidant (m^2^ s^–1^). Values for *D*_*x*(*p*)_ used in this study can be found in Table S2.

For the mixed regime, Wilson et al.^29^ expressed γ_rxn_ as a function of a bulk reaction rate constant (*k*_2_) and *k*_transport_ (1/τ_p,ox,diff_), which reflects the transport of the oxidant within the particle, where τ_p,ox,diff_ is computed using the in-particle diffusivity of the oxidant

10 $$\gamma_{rxn}=\frac{4aHRT\left[Y\right]}{3w}\left[\frac{k_{2}k_{transport}}{k_{2}\left[Y\right]+k_{transport}}\right]$$

This approximation is valid for droplet sizes >1 μm, which is true of all of all the single droplet experiments considered in this study, but may break down for accumulation-mode aerosol.

In a closed system, assuming that the amount of S­(IV) lost directly correlates to the amount of S­(VI) produced, we represent the concentration of S­(VI) in the system

11 $$\left[S\left(IV\right)\right]\left(t\right)={\left[S\left(IV\right)\right]}_{0}e^{-1/\tau_{sulfate}t}$$

For the reaction limited regime, the overall time scale of sulfate formation is expressed as

12 $$\frac{1}{\tau_{sulfate}}=\frac{3RTn_{x\left(g\right)}\beta }{a}+HRTn_{x\left(g\right)}k_{2}$$

This formulation captures the observed size dependence and moderate reaction rates from laboratory experiments (Figures 1 and 2) as well as the existence of a critical “tipping point” size above which bulk reactions dominate.^16,18^

To determine *k*_2_ and β, experimental data sets are modeled following the method outlined in Gong et al.^16^ Monte Carlo (MCMC) sampling is used to generate 10,000 random samples across a range of potential β values (10^–10^ to 1) and *k*_2_ values (10^–6^ to 10^3^ M^–1^ s^–1^), and the weighted sum of squares between the derived concentration profile and the experimental data is minimized. We find that the experimental systems of interest are more sensitive to β than to *k*_2_ when *k*_2_ is small (i.e., 0–10 M^–1^ s^–1^). Due to this insensitivity, MCMC sampling is useful in narrowing down the range for *k*_2_ to within 2 orders of magnitude, but finding an exact value for *k*_2_ would require running MCMC with a prohibitive number of samples. Next, we use the β range from the model output to compute a range for γ_*s*,*0*_. The tipping point radius, defined as the size below which surface kinetics dominate, is taken from experimental data where available.^18^ At the tipping point, the time scales of the surface process and bulk process are equivalent, indicating a transition from surface to bulk kinetics, so equating the two terms in eq 12 using the determined values for β yields a narrow range on *k*_2_. In cases where the tipping point is not reported due to a lack of size dependent data, *k*_2_ is chosen to generate bulk reactive uptake coefficients (γ_rxn_) that closely resemble known values.

For transport-limited systems

13 $$\frac{1}{\tau_{sulfate}}=\frac{3RTn_{x\left(g\right)}H\sqrt{D_{x\left(p\right)}k_{2}}}{2a\sqrt{{\left[Y\right]}_{0}}}$$

In transport-limited systems, kinetics are size-dependent not because of an interfacial process, but because the oxidant only penetrates the droplet a short distance before reaction, i.e., the diffuso-reactive length is short.^2,23^ Because there is one parameter of interest (*k*_2_), eq 13 is fit with a nonlinear least-squares method using the Python curvefit­() library.

Computing the time scale of formation is more informative than computing the rate of sulfate production, which is dependent on the concentration of S­(IV) in the system at a given time, and thus exhibits its own time dependence in a closed system. A flowchart explaining these two limits and the modeling process in these cases can be found in the Supporting Information (Figure S1).

We show in the following sections that this model and parametrization technique, which together we refer to as CHAI, is applicable to the systems mentioned above, and can also be modified to extend to other reactive systems including NO_2_^–^ oxidation and organic oxidation in the presence of O_3_.

## 3 Results and Discussion

We applied CHAI to analyze experimental data sets from the literature, including S­(IV) oxidation, and oxidation of particle-phase NO_2_^–^ and organics by O_3_.

### 3.1 S­(IV) Oxidation in Single Droplet Systems: Uncatalyzed and TMI Catalyzed Oxidation

Using CHAI and the experimental data from Li et al.,^18^ we determine β for uncatalyzed and TMI-catalyzed S­(IV) oxidation by O_2_ at different pH and RH conditions (Figure 4, Table 1).^16,18^ Li et al. reported the critical radius for each of these systems, allowing direct inference of *k*_2_. They estimated [S­(IV)] in the droplet to fall between 2.6 and 3.7 M using E-AIM and the hygroscopicity of S­(VI). Note that although E-AIM is formulated for sulfate, the ion pair parameters for activity coefficient estimation are expected to be similar for SO_3_^–2^ to within 5–10%.^38^ The concentration and time scale profiles are replicated using eq 12 (Figure 4). For each oxidant system, we find that CHAI represents the experimental data well, with R^2^ values ranging from 0.90 to 0.99 (Table 1).

**Figure 4:**
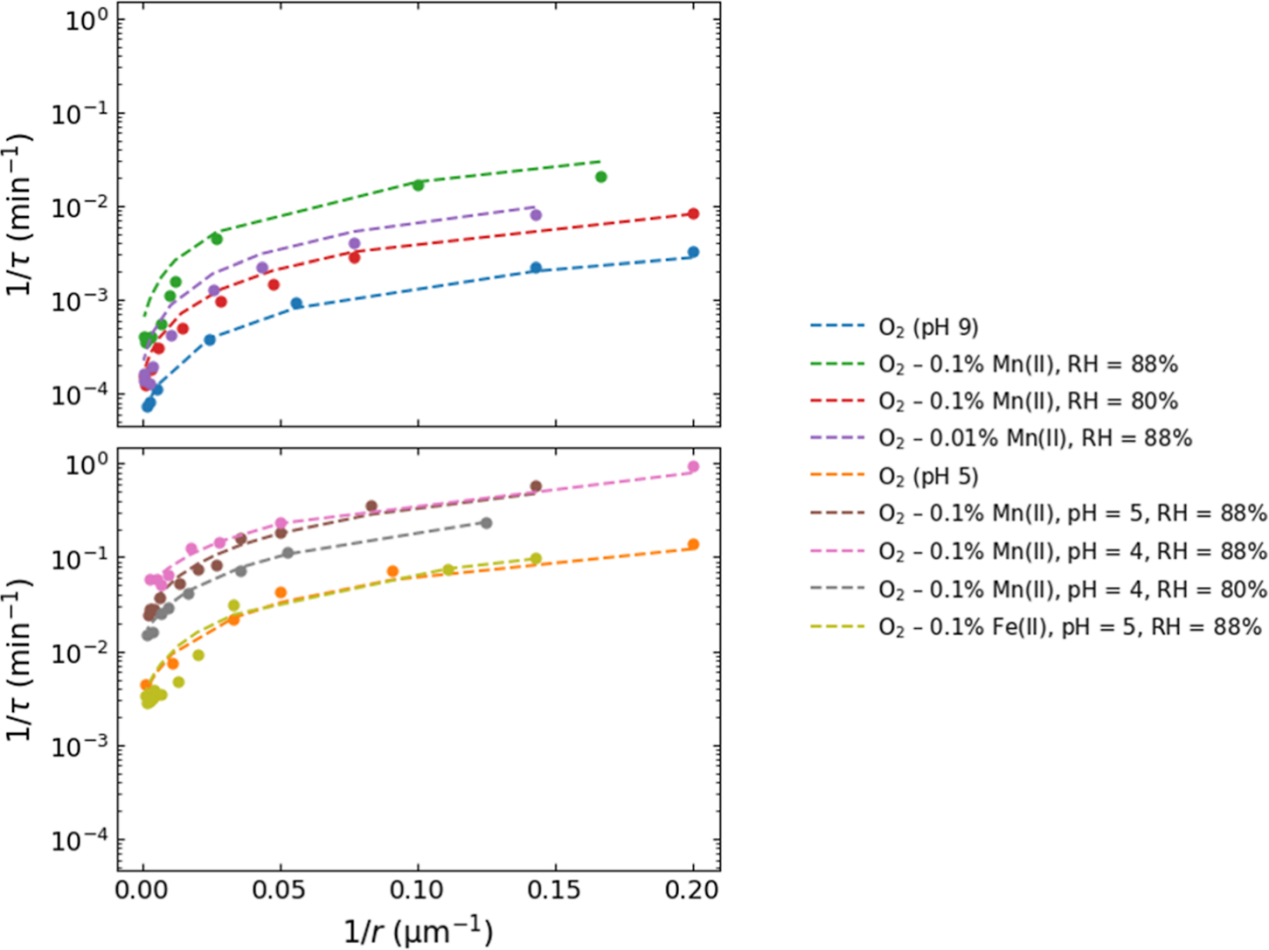
Sulfate formation time scale vs radius profiles for representative experiments from Li et al.^18^ These experiments include uncatalyzed O_2_ oxidation, Mn­(II) catalyzed O_2_ oxidation and Fe­(II) catalyzed O_2_ oxidation at (a) pH 9 and (b) pH 4–5.

**Table 1:** Inferred Surface and Bulk Parameters and Model-Measurement Agreement (R^2^ and Normalized RMSE (RMSE^N^)) for Uncatalyzed and TMI-Catalyzed Oxidation. Experiments are From Li et al.^18^

experiment	pH	RH	R^2^	RMSE^N^	β (m atm^–1^ s^–1^)	*k*_2_ (M^–1^ s^–1^)
O_2_	9	87	0.959	0.226	3.6 × 10^–10^ (+4.5 × 10^–10^/-3.4 × 10^–10^)	4.0 × 10^–3^ (+1.1 × 10^–3^/-8.9 × 10^–4^)
O_2_	5	86	0.948	0.269	1.6 × 10^–8^ (+3.0 × 10^–10^/-5.0 × 10^–10^)	1.7 × 10^–1^ (+3.9 × 10^–2^/-3.1 × 10^–2^)
O_2_ – 0.1% Mn(II)	9	88	0.900	0.463	4.6 × 10^–9^ (+1.2 × 10^–9^/-4.2 × 10^–10^)	3.3 × 10^–2^ (+1.5 × 10^–2^/-1.0 × 10^–2^)
O_2_ – 0.1% Mn(II)	9	80	0.987	0.162	1.1 × 10^–9^ (+5.7 × 10^–10^/-6.9 × 10^–10^)	9.3 × 10^–3^ (+2.9 × 10^–3^/-2.2 × 10^–3^)
O_2_ – 0.01% Mn(II)	9	88	0.936	0.311	1.8 × 10^–9^ (+1.1 × 10^–10^/-9.2 × 10^–10^)	1.2 × 10^–2^ (+2.6 × 10^–3^/-2.2 × 10^–3^)
O_2_ – 0.1% Mn(II)	5	88	0.900	0.330	8.4 × 10^–8^ (+5.0 × 10^–10^/-9.0 × 10^–10^)	1.2 (+1.6 × 10^–1^/-1.4 × 10^–1^)
O_2_ – 0.1% Mn(II)	4	88	0.952	0.269	9.9 × 10^–8^ (+5.0 × 10^–10^/-5.0 × 10^–10^)	2.5 (+7.8 × 10^–1^/-6.0 × 10^–1^)
O_2_ – 0.1% Mn(II)	4	80	0.997	0.055	4.6 × 10^–8^ (+1.2 × 10^–9^/-1.5 × 10^–9^)	8.5 × 10^–1^ (+4.7 × 10^–1^/-3.0 × 10^–1^)
O_2_ – 0.1% Fe(II)	5	84	0.984	0.168	1.7 × 10^–8^ (+3.0 × 10^–10^/-1.4 × 10^–9^)	1.8 × 10^–1^ (+1.0 × 10^–1^/-6.5 × 10^–2^)

**Table 2:** Inferred Surface and Bulk Parameters and Model-Measurement Agreement (*R* ^2^ and Normalized RMSE (RMSE^N^)) for H_2_O_2_ Oxidation. Experiments are From Li et al.^18^

experiment	pH	RH	*R* ^2^	RMSE^N^	β (m atm^–1^ s^–1^)	*k*_2_ (M^–1^ s^–1^)
H_2_O_2_ (5 ppm)	9	82	0.919	0.357	3.4 × 10^–4^ (+3.6 × 10^–4^/-2.9 × 10^–4^)	2.9 × 10^–5^ (+1.1 × 10^–5^/-7.9 × 10^–6^)
H_2_O_2_ (5 ppm)	5	82	0.951	0.279	2.4 × 10^–3^ (+8.9 × 10^–4^/-3.4 × 10^–4^)	9.4 × 10^–4^ (+2.2 × 10^–4^/-1.8 × 10^–4^)

For each of the uncatalyzed and TMI-catalyzed S­(IV) oxidation systems, the γ_*s*,*0*_ values are relatively small (<10^–2^), and decrease with decreasing [S­(IV)], consistent with the idea that γ_*s*,*0*_ is an upper limit for the surface uptake coefficient in closed systems, which may not be the case in systems where S­(IV) is continuously replenished from the gas phase. The relatively small uptake coefficients for this system are indicative of an efficient but not particularly fast surface reaction, consistent with our previous analysis of uncatalyzed S­(IV) oxidation by O_2_.^16^

As shown in Figure 4, uncatalyzed and TMI-catalyzed S­(IV) oxidation (with O_2_) are both accelerated in more acidic environments, with timesales that are approximately an order of magnitude smaller at pH 5 vs pH 9, consistent with observations of an “interfacial acceleration” in highly acidic particles (pH ≤ 3).^17,39^ The surface parameters and oxidation rates we report are consistent with those we previously derived for uncatalyzed sulfite oxidation based on the experimental data of Gong et al.^16^ (β = 8.42 × 10^–10^ (+8.30 × 10^–11^/–1.55 × 10^–10^) m atm^–1^ s^–1^, and *k*_2_ = 9.43 × 10^–3^(+2.26 × 10^–3^/–2.38 × 10^–3^) M^–1^s^–1^). As discussed in that paper, the *k*_2_ values correspond to faster rates than the rate parameters derived by Li et al., likely due to differences in our analytical and modeling approaches, but they are are consistent with pseudo-first-order rate constants reported in other studies of S­(IV) oxidation in bulk systems (Table S1).^40,41^

As shown in Figure 3, our initial time scale analysis suggested that some systems including Mn­(II)-catalyzed S­(IV) oxidation could fall in a mixed regime where reaction and diffusion are of similar importance. To further investigate this possibility, we have equated the *k*_2_ values calculated in this section assuming no transport limitation with the bracketed term in eq 10, and inferred the possible contribution of *k*_transport_ to the observed bulk reaction, for each system treated as reaction limited in this study. The results, shown in Figure S2, confirm that these systems are reaction limited and the treatment here is valid.

#### 3.1.1 S­(IV) Oxidation in Single Droplet Systems: H_2_O_2_, NO_2_, and O_3_ Oxidation

H_2_O_2_ oxidation, one of the dominant channels for S­(IV) oxidation in cloudwater,^15^ may also be important in smaller particles, with reported acceleration factors (compared to cloudwater) from aerosol flow reactor experiments of 1.5 due to increases in ionic strength from 2 to 6.5 molal, and an acceleration factor of 51 at 14 molal, which is typical of atmospheric aerosols.^32^

Our analysis in Section 2 (Figure 3) suggests that this system may be transport-limited at pH 5. However, the *k*_2_ necessary to replicate the experimental time scales reported by Li et al.^18^ using eq 13 (Figure 5) is 6.23 × 10^–5^ M^–1^ s^–1^ (+4.2 × 10^–6^/-7.3 × 10^–6^), several orders of magnitude lower than the value reported by Hoffmann and Calvert^37^ for bulk solutions, which was used for our analysis in Section 2. As noted previously, bulk reaction rates in aerosol may differ considerably from dilute solutions due to, i.e., ionic strength effects. For this *k*_2_, the system would necessarily be reaction limited. Therefore, we repeat the analysis using eq 12 (Figure 6). β is 2.4 × 10^–3^ m atm^–1^ s^–1^ at pH 5, which is approximately 5 orders of magnitude larger than β for the corresponding uncatalyzed oxidation experiment (Table 3). A high β value suggests that the interfacial process significantly impacts the kinetics. β comprises parameters which represent both the surface reaction rate coefficient and the density of reactant at the interface, so trends in β do not allow us to decouple rate vs concentration effects for rate enhancement at the interface. However, we also note that the atmospheric concentrations of O_2_ are more than 9 orders of magnitude higher than those of H_2_O_2_, and therefore the overall rates of these processes may be competitive. The pH sensitivity of the H_2_O_2_ pathway, taken as the enhancement in the bulk rate between pH 9 and pH 5, is smaller (32.4) than the enhancement in the uncatalyzed system (44.0). Our reported *k*_2_ value at pH 9 (2.91 × 10^–5^) is significantly lower than the *k*_2_ values in literature, pointing to the importance of future work that targets this system.^27^ Additionally, the data of Li et al. for H_2_O_2_ oxidation, which we model here, exhibits a much less significant pH dependence than Hoffmann and Calvert’s parametrization for this process in bulk solutions, suggesting that future work should also further examine the pH-dependence of the H_2_O_2_ system in droplets and aerosols.

**Figure 5:**
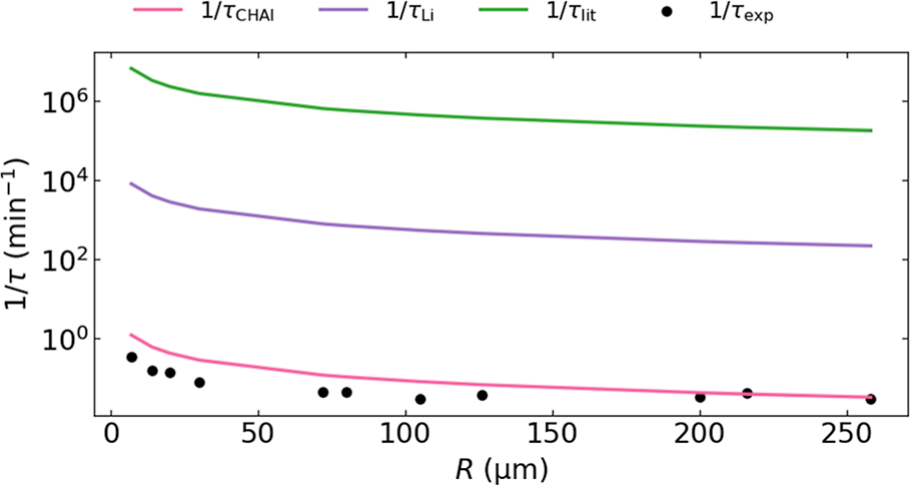
H_2_O_2_ oxidation at pH 5 parametrized as a fast bulk process, limited by comparatively slower diffusion. Various *k*_2_ values (Hoffmann and Calvert,^37^ Li et al.,^18^ transport limited CHAI output) are tested.

**Figure 6:**
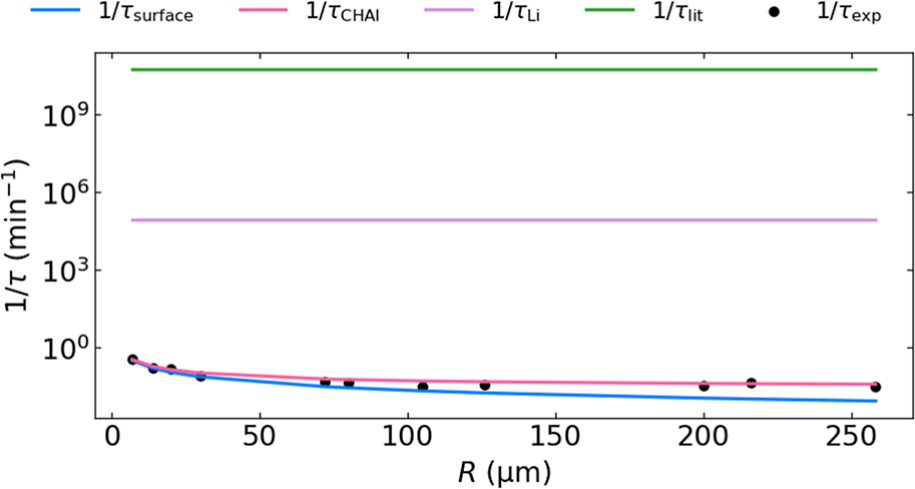
H_2_O_2_ oxidation at pH 5 parametrized as a slow bulk process with an efficient surface process. Various *k*_2_ values (Hoffmann and Calvert,^37^ Li et al.,^18^ reaction-limited CHAI output) are tested. The time scale of the surface process from CHAI is also shown.

**Table 3:** Inferred Bulk Rate Constant and Model-Measurement Agreement (R^2^ and Normalized RMSE (RMSE^N^)) for NO_2_ Oxidation and O_3_ Oxidation. Experiments are from Li et al.^18^

experiment	pH	RH	R^2^	RMSE^N^	*k*_2_ (M^–1^ s^–1^)
NO_2_ (5 ppm)	5	86	0.901	0.322	1.36 × 10^9^ (+1.40 × 10^8^/-2.00 × 10^8^)
O_3_ (1 ppm)	9	85	0.969	0.212	7.42 × 10^9^ (+3.20 × 10^8^/-3.30 × 10^8^)
O_3_ (1 ppm)	5	86	0.944	0.293	2.30 × 10^10^ (+3.70 × 10^9^/-3.70 × 10^9^)

The rate of NO_2_ oxidation of S­(IV) in bulk solutions is known to decrease with decreasing pH, potentially limiting its environmental relevance,^15,42^ but we note Li et al. observe the opposite trend. We model the data at pH 5, where the rate constants reported by Li et al. are more comparable to rate constants from literature and conditions are more environmentally relevant. The system is transport limited (Figure 7). Fitting the experimental data from Li et al. using eq 13 to determine *k*_2_ yields a value of 1.36 × 10^9^ M^–1^ s^–1^ (+1.40 × 10^8^ M^–1^ s^–1^/-2.00 × 10^8^ M^–1^ s^–1^). While this value is smaller than the value calculated by Liu et al.^33^ from their aerosol chamber data assuming uniform mixing, there is significant uncertainty associated with this rate constant in the bulk.^15,33^

**Figure 7:**
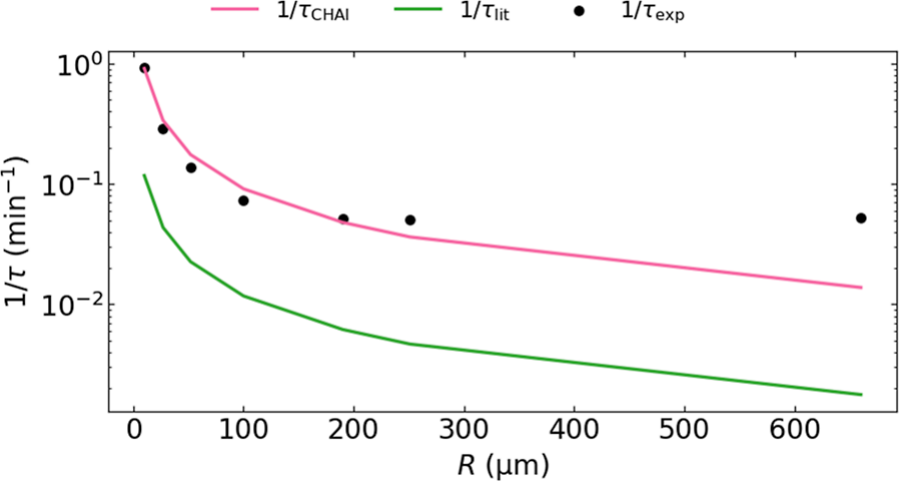
NO_2_ oxidation at pH 5 is parametrized as a transport limited bulk reaction. The CHAI-derived parametrization is compared to a fast bulk/transport-limited parametrization using the *k*_2_ reported by Liu et al.^33^

Similar to NO_2,_ the rate of O_3_ oxidation of S­(IV) in bulk solutions increases with increasing pH, although the overall faster rate increases its environmental relevance.^15,43^ Again, Li et al. observed a different trend in droplets, and our CHAI-derived parametrization reflects that. Based on Figure 3 we expect τ_bulk_ < τ_p,ox,diff_ at pH 9, and possibly a mixed regime at pH 5. Fitting the experimental data using eq 13 to determine *k*_2_ yields values of 7.42 × 10^9^ M^–1^ s^–1^ (+3.20 × 10^8^ M^–1^ s^–1^/-3.30 × 10^8^ M^–1^ s^–1^) at pH 9 and 2.30 × 10^10^ M^–1^ s^–1^ (+3.70 × 10^9^ M^–1^ s^–1^/-3.70 × 10^9^ M^–1^ s^–1^) at pH 5 (Figure 8). These rate constants are higher than the values reported for bulk solutions by Hoffmann and Calvert^37^ which were used to generate Figure 3. Reevaluating τ_bulk_ using these higher *k*_2_ values confirms that the O_3_ oxidation system is transport limited.

**Figure 8:**
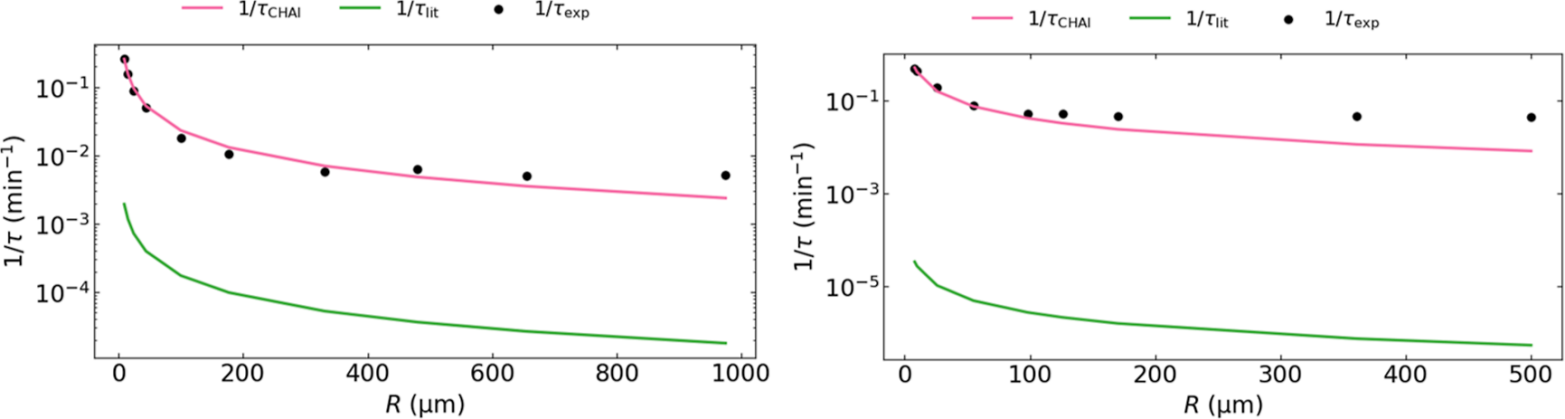
O_3_ oxidation is parametrized as a fast bulk process, limited by comparatively slower diffusion (L: pH 9, R: pH 5). The CHAI-derived parametrization is compared to a fast bulk/transport-limited parametrization using the *k*_2_ from Hoffmann and Calvert.^37^

A summary of the rate constants and their uncertainties for these three cases is given in Table 4.

**Table 4:** Reported γ_*s*_ and *γ*_*rxn*_ for Representative Ozonolysis Experiments with Trans-Aconitic Acid (AA), Maleic Acid (MA), Nitrite (NO_2_^–^), Fumarate (FA), and Ascorbic Acid (AscA) from Willis and Wilson^31^ and Wilson et al.,^29^ as Well as the Corresponding Values for Each Experiment Derived from CHAI

experiment	a (μm)	[O_3_] (ppm)	γ_*s*_(CHAI)	γ_*s*_ (REF)	γ_rxn_ (CHAI)	γ_rxn_ (REF)	γ_overall_(CHAI)	γ_overall_ (REF)	ref
AA (3.2 M)	9.23	58.4	3.02 × 10^–7^	4.67 × 10^–7^	2.14 × 10^–5^	3.54 × 10^–6^	2.19 × 10^–6^	4.01 × 10^–6^	31
MA (7.4 M)	4.59	38	2.08 × 10^–7^	2.55 × 10^–7^	1.15 × 10^–5^	3.75 × 10^–6^	1.17 × 10^–5^	4.01 × 10^–6^	31
NO_2_^–^ (0.2 M)	5.75	12	3.28 × 10^–5^	2.55 × 10^–5^	5.79 × 10^–6^	4.88 × 10^–6^	3.86 × 10^–5^	3.04 × 10^–5^	31
FA (0.086 M)	4.5	1	2.50 × 10^–5^	1.7 × 10^–5^	7.31 × 10^–6^	6.1 × 10^–6^	3.24 × 10^–5^	2.30 × 10^–5^	29
AscA (3.55 M)	3.44	42	8.38 × 10^–5^	6.4 × 10^–5^	1.01 × 10^–5^	8.1 × 10^–6^	9.39 × 10^–5^	7.20 × 10^–5^	29

### 3.2 S­(IV) Oxidation in Aerosol Reactor Systems

CHAI may be used to reconcile observations from size dependent single-droplet experiments and aerosol reactor studies. These experimental approaches differ in several ways, including time scales, experimental conditions, particle size, and the fact that it is an ensemble of particle sizes studied, thus making direct intercomparison of their results without a suitable model challenging.

#### 3.2.1 NO_2_ Oxidation

Liu and Abbatt^33^ showed that, for submicron deliquesced aerosol particles at pH 4–5, the effective rate constant for the reaction of NO_2_ with S­(IV) is 3 orders of magnitude larger than its value in dilute solutions. They hypothesized that interfacial chemistry drives fast kinetics, but, in the absence of size-dependent kinetic information to confirm this hypothesis, they opted to model NO_2_ oxidation of SO_3_^–2^ in their experiments as a fast bulk reaction

14 $$\frac{d\left[SO_{4}^{2-}\right]}{dt}=k_{\exp}K_{a1}H_{SO_{2}}P_{SO_{2}}H_{NO_{2}}P_{NO_{2}}$$

where *k*_exp_ (M^–2^ s^–1^) encompasses the reaction rate constants for both NO_2_+HSO_3_^–^ and NO_2_+SO_3_^2–^, *K*_a1_ is the thermodynamic dissociation constant of H_2_SO_3_ (1.3 × 10^–2^ M), and *H*_SO2_, *P*_SO2_, *H*_NO2_, and *P*_NO2_ are the Henry’s law constants and partial pressures of SO_2_ and NO_2_, respectively. Liu and Abbatt set *k*_exp_ *K*_a1_, which has units of M^–1^ s^–1^, to be the overall observed second order rate constant in their system. For this system, *k*_exp_ typically ranges between 10^12^ and 10^14^ M^–2^ s^–1^ for ammonium nitrate seed aerosol, with a corresponding *k*_ex*p*_ *K*_a1_ value between 10^10^ and 10^12^ M^–1^ s^–1^. Because their experimental data were not size dependent, the rate expressed by eq 14 is size independent.

We seek to determine whether the aerosol flow reactor results of Liu and Abbatt, and their proposed size independent model, can be reconciled with the single droplet results of Li et al.^18^ To this end, we applied CHAI, with the parametrizations derived from the results of Li et al. in the previous section (Table 3), to model an experiment of interest from Liu and Abbatt (AN seed aerosol, RH 64%, 10.4 ppb SO_2_, 655 ppb NO_2_). The reported pH range is between 3.8 and 5.2, and they note that the presence of organics in the system makes this estimate uncertain. The most similar experiment from Li et al. was the NO_2_ oxidation experiment at pH 5, which yielded *k*_*2*_ = 1.36 × 10^9^ M^–1^ s^–1^ when modeled as a transport limited system using CHAI. However, the low [Y] and small particle radius places Liu and Abbatt’s experiment in a reaction limited regime (*k*_*2*_[Y] ≪ *k*_transport_). Therefore, to model this experiment using CHAI, we apply eq 12. using *k*_*2*_ = 1.36 × 10^9^ M^–1^ s^–1^ and β = 0.

Using the mass distribution for sulfate from Liu and Abbatt, we find that the peak of the corresponding surface area weighted distribution is ∼200 nm (Figure S3). We determine the rate of sulfate formation (d­[SO_4_^2–^]/dt) based on the portion of the kinetics time series after the addition of NO_2_, when P_SO2_ is at roughly steady state (10.4 ppb). Liu and Abbatt report a *k*_exp_ value of 6.9 × 10^12^ M^–2^ s^–1^ for this system, and a corresponding sulfate formation rate of 9 × 10^–6^ M s^–1^. The effective Henry’s law constant is assumed to be 100 M/atm, which corresponds to a pH of ∼ 3.8.

We compare results from eq 14 to the reaction-limited case from CHAI (eq 12) and find that the rates are comparable at ∼ 200 nm — if the rate constant is held at the value at pH 5 (1.36 × 10^–6^ M^–1^ s^–1^), the rate of sulfate formation (d­[SO_4_^2–^]/dt) is 1.11 × 10^–5^ M s^–1^, which is comparable to the d­[SO_4_^2–^]/dt value of 9 × 10^–6^ M s^–1^ reported by Liu and Abbatt.^33^ This agreement suggests that interfacial processing is not dominant for NO_2_ oxidation of S­(IV), the rate enhancement observed by Liu and Abbatt for the aerosol phase was due to bulk chemistry effects such as ionic strength, and the size-independent parametrization is valid for low S­(IV) concentrations and small droplet sizes.

#### 3.2.2 H_2_O_2_ Oxidation

In the case of H_2_O_2_ oxidation, Liu et al.^32^ utilized a flow reactor to analyze the oxidation of dissolved SO_2_ by hydrogen peroxide (H_2_O_2_). Again, they observe enhanced kinetics in the aerosol phase compared to bulk rates. They attributed this to ionic strength effects. They tested a variety of seed aerosols, including a mixture of NaCl, sodium bimalonate, and sodium malonate (20:1:1); the aerosols were assumed to be entirely NaCl, and on this basis their size distribution was inferred32 and thermodynamic calculations were performed.^44^ Similar to the NO_2_ oxidation system, Liu et al. proposed that the rate of sulfate formation can be represented using a size-independent bulk scheme

15 $$\frac{d\left[SO_{4}^{2-}\right]}{dt}=\left({k+k}_{HX}\left[HX\right]/\left[H^{+}\right]\right)K_{a1}H_{SO_{2}}P_{SO_{2}}H_{H_{2}O_{2}}P_{H_{2}O_{2}}$$

where *k* is the reaction rate constant (M^–2^ s^–1^), *k*_*HX*_ is the rate constant for the acid catalysis mechanism (M^–2^ s^–1^), *H*_H2O2_ and *P*_H2O2_ are the Henry’s law constant and partial pressures of H_2_O_2_, and the other parameters are as defined previously.

Liu et al. state that the partial pressures of SO_2_ and H_2_O_2_ were kept constant and in excess in each experiment so that the sulfate that forms is solely dependent on the reaction time — thus, we can assume that the initial mixing ratios of SO_2_ and H_2_O_2_ are constant throughout the experiment.^32^ Given an initial SO_2_ concentration of 343 ppb, an H_2_O_2_ concentration of 94 ppb, and a pH 2.3–5.8 (uncertain due to discrepancies in the thermodynamic models used and the presence of organics in the system), Liu et al. extrapolate a measured SO_4_^2–^ formation rate of 0.0149 molal/s. Using their observed formation rate, we can derive (*k* + *k*_*HX*_[HX]/[H+])*K*_*a1*_ to be 2.50 × 10^2^ M^–1^ s^–1^ using an effective Henry’s law constant of 18,000 (corresponding to pH around 5.5).

We compare eq 15 with the output from CHAI for reaction-limited H_2_O_2_ oxidation at pH = 5 (Table 3, β = 2.4 × 10^–3^ m atm^–1^ s^–1^ *k*_*2*_ = 9.42 × 10^–4^ M^–1^ s^–1^). We find that at 0.02 μm, which is the peak of the surface area weighted distribution, the sulfate production rate is in agreement between the two models (0.0128 molal/s) (Figure 9). The *x*-axis is presented on a log scale to make the size range of interest (between 0.02 and 0.03 μm) visible. Again, we can estimate the difference in predicted sulfate production rate for an atmospherically relevant aerosol size distribution using the fast bulk rate vs the size-dependent CHAI scheme, and find that the total amount of sulfate produced between the two reaction schemes differs by a factor of 160, with the fast bulk scheme producing more sulfate across the distribution — 0.0265 μg m^–3^ h^–1^ air in the fast bulk scheme vs 0.000165 μg m^–3^ h^–1^ air in the CHAI scheme. The overlap between the CHAI derived parametrization and the fast bulk scheme occurs at 0.02 μm, which is the left tail end of the atmospherically relevant size distribution, and the fast bulk scheme overestimates rates across the remainder of the distribution. This illustrates the importance of accounting for size-dependent kinetics in atmospheric models for systems with significant interfacial chemistry.

**Figure 9:**
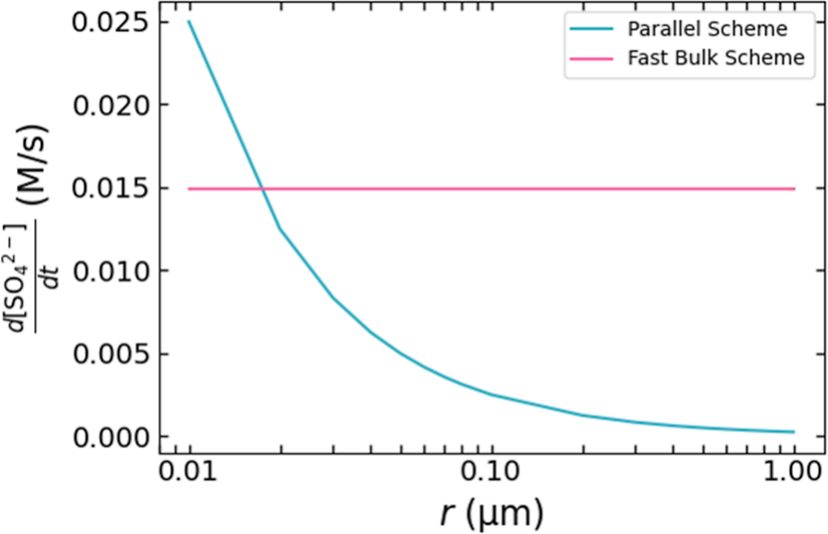
Rates of sulfate formation (d­[SO_4_^2–^]/dt) via H_2_O_2_ oxidation for the size independent scheme of Liu et al.^32^ and CHAI, using a parametrization derived from the single droplet data of Li et al.^18^

### 3.3 Applications to Other Multiphase Reactive Systems

Wilson and co-workers^29,31^ modeled experimental data for the ozonolysis of sodium nitrite,^45^ *trans*-aconitic acid,^31^ maleic acid,^46^ ascorbic acid,^47^ and fumarate,^48^ and derived surface and bulk reactive uptake coefficients for these systems using a stochastic kinetic model that represents the droplet as having microcompartments (including a surface and bulk region), and physically constrains the parameters describing reactions, partitioning, and diffusive mass transfer with experimental data (Table 2). Here we investigate whether CHAI, a simple two-parameter model, is a suitable alternative approach to describe these systems. We present the analysis for NO_2_^–^, maleic acid, and ascorbic acid in this section.

Using the CHAI approach we derive β, and subsequently γ_*s*_, from the experimental data sets, and constrain *k*_2_ based on bulk observations. As explained by Wilson et al., many of these reactions fall in the mixed transport-reaction regime, and γ_*rxn*_ should be parametrized by eq 10; because of the nonlinear dependence on [Y], the rate law cannot be directly integrated to obtain a simple closed form expression for the time scale of product formation for mixed regimes.^29^ In these cases, we apply CHAI with a slightly different approach: Given *k*_2_, β is derived using the MCMC algorithm described in Section 2. β *is* used to compute γ_*s*_ (eq 5), which is compared to the values reported by Wilson et al.^29^ or Willis and Wilson.^31^ *k*_transport_ can be computed following eq 16, with a constant prefactor in its formulation for consistency with Wilson et al., and is valid for droplets >1 μm in radius.^29^

16 $$k_{transport}=\frac{2D_{x\left(p\right)}}{{\left(\frac{a}{3}\right)}^{2}}$$

Because *k*_2_ is reported for these systems, and *k*_transport_ can be calculated, we can find a value of γ_*rxn*_ from eq 10., and compute γ_overall_ following eq 4. The agreement between our γ_overall_ values and values from Wilson et al.^29^ or Willis and Wilson^31^ (Table 4) shows that, in a mixed reaction-transport regime, if a bulk rate constant is known, it is possible to calculate a γ_overall_ using CHAI, which would be suitable for implementation in a large-scale model. As noted in Section 2, the MCMC algorithm for determining β is relatively insensitive to the choice of *k*_2_.

#### 3.3.1 NO_2_^–^ Ozonolysis

Hunt et al.^45^ monitored NO_2_^–^ decay in optically trapped single particles exposed to ozone using Raman spectroscopy, and observed accelerated kinetics in droplets. They modeled their experimental results with a scheme involving liquid phase diffusion and a bulk reaction, and no surface reaction. Willis and Wilson used a bulk rate consistent with measurements from Hunt et al. to show that bulk kinetics yield NO_2_^–^ decay that is slower than the experimental observations, and suggested that a more complex model including surface reactions is required.

Willis and Wilson assumed that the interfacial rate constant for oxidation of nitrite is the same as that in the bulk. Using CHAI, we derive β from data across the 10 experiments of Hunt et al.^45^ We find that β = 6.05 × 10^–4^ (−6 × 10^–6^/+2.1 × 10^–6^) m atm^–1^ s^–1^, which correlates to a γ_*s*_ of 3.28 × 10^–5^ (−6 × 10^–7^/+2 × 10^–7^) (eq 5). The CHAI derived γ_*s*_ is on the same order as the surface uptake coefficient reported by Willis and Wilson (γ_s_ = 2.55 × 10^–5^).^31^

Next, we use the value of *k*_2_ from bulk experiments and *k*_transport_ for the experiment of interest (r = 5.75 μm, [NO_2_^–^] = 0.2 M, [O_3_] = 12 ppm) to find γ_rxn_ (eq 10). We find that for a *k*_2_ value of 10^5^ M^–1^ s^–1^ and a *k*_transport_ value of 1034 s^–1^, γ_rxn_ = 5.79 × 10^–6^, which is comparable to the bulk uptake coefficient reported by Willis and Wilson (4.88 × 10^–6^).^31^ Both the CHAI-derived scheme and the parametrization by Wilson et al.^29^ reflect the fact that this is a surface-dominated process, and the reported overall uptake coefficients are comparable (Table 4). We note that all the experiments in this study fall in roughly the same size range (between 4 and 6 μm) so there is not sufficient data to fully explore the size dependence of this system.

#### 3.3.2 Maleic Acid Ozonolysis

Willis and Wilson simulated experiments by Dennis-Smither et al.^46^ who observed the loss of particle-phase maleic acid (MA) in aqueous droplets via O_3_ reaction using aerosol optical tweezers and cavity enhanced Raman spectroscopy over a range of relative humidities. Dennis-Smither et al. found that exposing MA droplets to ozone forms products with a range of volatilities, and that reactive uptake coefficients show a weak dependence on ozone concentration, but do not depend on RH.

Willis and Wilson used their microcompartment model, accounting for surface and bulk reaction as well as the evaporation of MA from the droplet over the course of the reaction.^31^ They compared their model results to a portion of the data from Dennis-Smither et al., recorded at RH < 73%, and neglected the higher RH experiments, during which droplets grew during ozonolysis due to an increase in hygroscopicity. Willis and Wilson concluded that MA ozonolysis is similar to the AA ozonolysis system, in that it is dominated by bulk phase kinetics. Following the analysis from above, we fit experimental data for the RH < 73% experiments from Dennis-Smither et al., and obtain β = 1.04 × 10^–7^ (−2 × 10^–9^/+8 × 10^–9^) m atm^–1^ s^–1^, and γ_*s*_ = 2.08 × 10^–7^ (−2 × 10^–9^/+1.6 × 10^–8^). Using the parametrization in eq 10 to describe the system (a mixed reaction-transport regime) with a *k*_2_ value of ∼ 10^3^ M^–1^ s^–1^ and a *k*_transport_ = 1623 s^–1^ yields γ_rxn_ = 3.43 × 10^–5^, which is roughly 10x larger than the γ_*rxn*_ reported by Wilson et al. (Table 4). This difference may be due in part to the fact that Willis and Wilson used a different *k*_2_ value which they derived from the experimental data. Recognizing that *k*_2_[*Y*] (7400 s^–1^) is larger than *k*_transport_ (1623 s^–1^), we also model this system as a transport-limited process (eq 13) and find that γ_*rxn*_ is 1.15 × 10^–5^, while γ_overall_ is 1.17 × 10^–5^, which are roughly 3x faster than the observed kinetics. We conclude that CHAI can provide insights into the relative importance of surface processes for systems like this, but a more complex model, as described by Wilson et al.,^29^ may be necessary to fully represent this chemistry.

#### 3.3.3 Ascorbic Acid Ozonolysis

Chang et al. investigated the reaction kinetics of Ascorbic acid (AscA) ozonolysis in single droplets with aerosol optical tweezers and Raman spectroscopy.^47^ They found that the kinetics exhibit a negative pH dependence, with a bulk reaction rate (*k*_*rxn*_) of 3.1 × 10^5^ M^–1^ s^–1^ at pH 2, and 1.2 × 10^7^ M^–1^ s^–1^ at pH 6, and concluded that the observed kinetics can be explained by liquid phase diffusion and a bulk reaction between AscA + O_3_. Wilson et al., using their microcompartment model and using an ozonolysis rate coefficient for AscA of *k*_2_ = 6.9 × 10^5^ M^–1^ s^–1^, reported that this reaction is better described by a surface reaction which accounts for >80% of the reactive processing, with minor contributions in the bulk droplet.^23^

Following Wilson and Prophet, we excluded data with longer reaction time scales (>2000 s) from our analysis. We derive β = 8.75 × 10^–5^ (−3 × 10^–7^/+6 × 10^–7^) m atm^–1^ s^–1^ and consequently γ_*s*_ = 8.38 × 10^–5^ (−3 × 10^–7^/+6 × 10^–7^). Using the parametrization in eq 10 to describe the system (a mixed reaction-transport regime) with a *k*_2_ value ∼ 10^5^ M^–1^ s^–1^ and a *k*_transport_ value of 2980 s^–1^ yields a γ_rxn_ value of 1.01 × 10^–5^, similar to the reported value of 8.1 × 10^–6^ for the experiment of interest (*a* = 3.44 μm, [AscA] = 3.55 M, [O_3_] = 42 ppm). The γ_overall_ values are comparable (9.39 × 10^–5^ in CHAI vs 7.2 × 10^–5^ in Wilson et al.), pointing to the utility of CHAI in determining the importance of the surface process and modeling systems with dominant interfacial reactions (Table 4).

## 4 Environmental Implications

Size-dependent reactive processes, including the ones discussed here, may be significant for air quality and climate, and they are not currently represented in large-scale atmospheric models. Following eq 4–8, they may be represented as reactive uptake processes with appropriate parametrizations of γ_obs_

17 $$\gamma_{obs}=\frac{4RT}{w}\left[Y\right]\left(\beta +\frac{Ha}{3}k_{2}\right)$$

For O_3_ and NO_2_ oxidation under environmental conditions, kinetics are not expected to be transport limited, so the same formulation applies, with β = 0. While higher in-particle reactant concentrations in laboratory studies can result in transport-limited kinetics for systems with faster bulk reactions, lower reactant concentrations under environmental conditions means a decrease in *k*_2_[Y], and the system may transition to the reaction limited regime. As discussed in Section 3.2.1, CHAI can then be applied in the slow-reaction limit (eq 12).

The rate of reactant loss from the gas phase is then expressed as

18 $$R_{loss}=\frac{n_{x\left(g\right)}wS_{a}\gamma_{obs}}{4}$$

where *S*_a_ is the particle surface area per volume of gas (cm^2^cm^–3^). The loss rate of particle-phase reactant is given by

19 $$R_{loss,p}=\frac{3n_{x\left(g\right)}w\gamma_{obs}}{4a}$$

Following eq 17, γ_obs_ is a function of the parameters *k*_2_ and β, as well as particle size, particle-phase reactant concentration [Y], and temperature. It is typical for large-scale atmospheric models to represent the aerosol using a fixed log–normal size distribution, with a relative humidity-dependent hygroscopic growth factor for each particle ‘type’ (e.g., sulfate-nitrate-ammonium in GEOS-Chem).^49–51^ Therefore, particle size, and therefore γ_obs_ is also a function of relative humidity. The procedure for determining [Y] will depend on the reaction family and the model architecture. For oxidation of S­(IV) species, the concentration of S­(IV) may be determined following Henry’s Law as follows

20 $$\left[S\left(IV\right)\right]=H_{\text{SO}2}P_{\text{SO}2}\left[1+\frac{K_{s1}}{\left[H+\right]}+\frac{K_{s1}K_{s2}}{{\left[H+\right]}^{2}}\right]$$

where

21 $$K_{s1}=0.13\exp\left(1960\left(\frac{1}{T}-\frac{1}{298}\right)\right)$$

and

22 $$K_{s2}=6.6\times 10^{-8}\exp\left(1500\left(\frac{1}{T}-\frac{1}{298}\right)\right)$$

Both *K*_s1_ and *K*_s2_ have units of M. For the Henry’s Law constant of SO_2_, H_SO2_

23 $$H_{\text{SO}2}=1.23\exp\left(-\frac{6.25}{R}\left(\frac{1}{298}-\frac{1}{T}\right)\right)$$

[H^+^] and aerosol liquid water content may be calculated using an aerosol thermodynamic model such as ISORROPIA^52^ or E-AIM.^44^

Tables 1–3 list our derived parameters for S­(IV) oxidation processes. The *k*_2_ and β parameters shown in Table 5 were selected as a subset of the entries in Tables 1–3 based on environmental modeling relevance. These parameters are valid for ∼pH 5 and deliquesced aerosol (derived from experimental data with RH > 82%). Aerosol pH is not expected to exceed 5,^53^ and pH ∼ 4.5 has been reported for Beijing haze episodes.^54^ As several of these processes are expected to be pH dependent, and there is ample evidence that the pH dependence of aerosol-phase S­(IV) oxidation processes differs from the bulk solution kinetics active in cloud droplets, more experimental data are needed so that parametrizations may be developed for pH < 5. No recommendation is made here for the O_2_–Fe­(II) system because the reported parameters were nearly identical to those for uncatalyzed O_2_ oxidation (Table 1). Although it is uncommon for large-scale models to track either Fe­(II) or Mn­(II) as aerosol components, given its possible importance for urban haze we include the parametrization for Mn­(II)-catalyzed oxidation here. “Mn­(II) 0.1%” in the experiments of Li et al.^18^ corresponds to ∼5 × 10^–3^ M Mn, which is consistent with Beijing haze conditions.^54^ In addition to the CHAI parameters, Table 5 also shows γ_obs_ and the sulfate production rate, calculated using environmental parameters for Beijing haze conditions (see Supporting Information for additional details).^42,54^ We note that the sulfate production rate estimations presented in Table 5 differ slightly from those calculated in Section 3.2 because here we use surface area weighted average particle radius and *S*_a_ from Wang et al.^42^ The estimated aerosol-phase sulfate production rate based on NO_2_ and O_3_ oxidation, as well as Mn-catalyzed S­(IV) oxidation by O_2_ is high, 1.2 × 10^2^ μg m^–3^ h^–1^ 17 μg m^–3^ h^–1^_,_ and 19 μg m^–3^ h^–1^, respectively, consistent with rapid sulfate formation during a haze event. The yield from uncatalyzed O_2_ oxidation is an order of magnitude slower, 3.2 μg m^–3^ h^–1^. The HOOH pathway is slow enough to be negligible under these conditions. This trend is contrary to expectations based on bulk solution kinetics, but consistent with environmental observations.^14,42,54^ Consistent with our calculations in Section 3.2, the estimated sulfate production rates in Table 5 for NO_2_ oxidation, which is not dominated by interfacial processes, are similar in order of magnitude with those of Liu and.

**Table 5:** Parameterization for Size-dependent aerosol Phase S­(IV) Oxidation at pH 5 Based on CHAI Analysis of Experimental data. Overall Reactive Uptake Coefficient and Sulfate Formation Rate Calculated for Each Pathway Assuming Beijing winter Haze Conditions.^28,32^

S(IV) oxidation pathway	β (m atm^–1^ s^–1^)	*k*_2_ (M^–1^ s^–1^)	γ_*obs*_	*R*_sulf_ (μg m^–3^ h^–1^)
O_2_	1.6 × 10^–8^ (+3.0 × 10^–10^/-5.0 × 10^–10^)	1.7 × 10^–1^ (+3.9 × 10^–2^/-3.1 × 10^–2^)	2.5 × 10^–12^	3.2
O_2_ – 0.1% Mn(II)	9.5 × 10^–8^ (±4.4 × 10^–10^)	1.25 (±0.15)	1.5 × 10^–11^	1.9 × 10^1^
H_2_O_2_	2.4 × 10^–3^ (+8.9 × 10^–4^/-3.4 × 10^–4^)	9.4 × 10^–4^ (+2.2 × 10^–4^/-1.8 × 10^–4^)	4.0 × 10^–7^	2.3 × 10^–5^
NO_2_	0	1.36 × 10^9^ (+1.40 × 10^8^/-2.00 × 10^8^)	3.5 × 10^–4^	1.2 × 10^2^
O_3_	0	2.3 × 10^10^ (±3.7 × 10^9^)	3.5 × 10^–3^	1.7 × 10^1^

Abbatt.^33^ However, including the size dependence for H_2_O_2_ results in a lower estimated sulfate production rate.

We note that the environmental conditions of many of the experimental studies modeled here represent significantly higher gas-phase and particle-phase reactant concentrations than typical for the atmosphere, which may limit their environmental relevance. The resistor model framework allows us to extend the model to atmospherically relevant conditions to understand the potential environmental impact of this chemistry. A lower particle-phase reactant (e.g., S­(IV)) concentration will result in a lower pseudo-first-order bulk reaction rate as well as reduced surface concentration of the reactant. This concentration, reflected in the concentration [Y] in eq 17, affects γ_obs_ linearly.

Lower gas phase concentrations (typically ∼ ppb for the gas-phase reactants considered other than O_2_) will result in lower overall collision rate, which will be represented in eqs 18 and 19. Gas-phase diffusion, the time scale for which depends on particle length scale and the mean free path of air but not gas-phase concentration, is not expected to be a factor for submicron aerosols. Likewise, the particle-phase diffusion time scale is not affected by concentration. Therefore, we conclude that the time scale analysis used in the development of the model holds, and the model may be extended to a range of atmospherically relevant reactant concentrations.

We have demonstrated using CHAI that the size dependence of reactive aerosol processes, such as S­(IV) oxidation in aqueous aerosols, can affect the overall observed kinetics for a polydisperse aerosol population and should be taken into consideration when these processes are represented in atmospheric chemistry and air quality models. The CHAI approach is useful for modeling these systems across size scales and for developing parametrizations for large-scale modeling. Given the likely significance of these processes and the marked difference between kinetic trends such as pH dependence in submicron aerosols vs bulk solutions, continued size-dependent experimental investigations of interfacial aerosol chemistry, especially at environmentally relevant aerosol pH, in-particle and gas-phase concentrations, are needed.

## Supplementary Material



## Notes

Details of each term in eq 1, pseudo-first order rate constants for S­(IV) → S­(VI) oxidation for dilute solutions, values of in-particle diffusivities for various oxidants, detailed descriptions of experiments from Li et al., flowchart describing two limits for CHAI, quantification of the influence of transport in a reaction limited regime, particle size distribution information for Liu et al., CHAI analysis of *trans*-acotinic acid and fumarate data, assumed conditions for environmental calculations (PDF)
