# Supplementary material for: Modeling and Parametrization of Size-Dependent Processes in Multiphase Aerosol Chemistry
Source: ACS EST Air. 2026 Jun 4;3(7):1733–44. doi: 10.1021/acsestair.5c00486 (PMC13366576; doi:10.1021/acsestair.5c00486)
Supplement: Supplementary file 1 [file ea5c00486_si_001.pdf]

Supporting Information for:

Modeling and parameterization of size-dependent processes in multiphase aerosol chemistry

Sandhya Sethuraman[1], Daniel M. Westervelt[2], Kedong Gong[3], Vicki H. Grassian[3], V. Faye McNeill[1,4,5*]

[1]Department of Chemical Engineering, Columbia University, New York, NY 10027, USA

[2]Lamont-Doherty Earth Observatory, Columbia University, Palisades, NY, 10964, USA

[3]Department of Chemistry, University of California San Diego, La Jolla, CA, 92093, USA

[4]Department of Earth and Environmental Sciences, Columbia University, New York, NY 10027, USA

[5]Climate School, Columbia University, New York, NY 10027, USA

*Corresponding Author. Email: vfm2103@columbia.edu

S1. Supplementary Equations

The functional forms of the terms in eq. (1) appear in the main text, but are also given as follows:

$$\gamma_s = \frac{4k_{2,s}H_sRTK_s[Y]}{w} \quad \text{(S1)}$$

Reaction-limited:

$$\gamma_{rxn} = \frac{4aHRT}{3w}k_2[Y] \quad \text{(S2a)}$$

Transport-limited:

$$\gamma_{rxn} = \frac{4HRT}{w}\sqrt{D_{x(p)}k_2[Y]} \quad \text{(S2b)}$$

Mixed regime:

$$\gamma_{rxn} = \frac{4aHRT[Y]}{3w}\left[\frac{k_2\, k_{transport}}{k_2\,[Y] + k_{transport}}\right] \quad \text{(S2c)}$$

$$\gamma_{diff} = \frac{Kn(1+Kn)}{0.75 + 0.283Kn} \quad \text{(S3)}$$

$$\gamma_{p,diff} = \frac{16D_y}{n_{x(g)}w}\frac{[Y]}{a} \quad \text{(S4)}$$

$$w = \sqrt{\frac{8k_BT}{\pi m}} \quad \text{(S5)}$$

In these equations, H is the Henry's law constant (M/atm), R is the gas constant (L atm mol^{-1} K^{-1}), T is the temperature (K), w is the mean thermal velocity, $n_{x(g)}$ is the concentration of the oxidant in the gas phase, and k_2 is the bulk rate constant.[1] In eq. S1, k_{2s}, H_s, and K_s are surface analogues of the bulk rate constant, Henry's law constant, and surface partitioning equilibrium constant, which form the parameter β.[1] In eq.

S3, *Kn* is the Knudsen number, defined as the mean free path divided by the particle radius. In eq. S4, D_y (m^2 s^{-1}) is the diffusivity of the aqueous phase species, in this case, S(IV), in the droplet.

The bulk reactive uptake coefficient is parameterized in three different regimes: eq. S2a represents the reaction limited regime, eq. S2b represents the transport limited regime, where $D_{x(p)}$ (m^2 s^{-1}) is the diffusivity of the gas-phase oxidant in the droplet, and eq. S2c represents the mixed regime (where both the bulk reaction and oxidant transport are important). $k_{transport}$ (s^{-1}) is defined as the rate of oxidant transport from the surface to the droplet to the interior.[2,3]

S2. Literature data, experimental conditions and fit statistics

Table S1. Values of the bulk pseudo-first order rate constant for S(IV) → S(VI) oxidation by O_2, H_2O_2, NO_2, and O_3 reported in literature.

Experiment	pH	RH	k_b (s^{-1})	Reference
O_2	9	87	1.50×10^{-5}	Radojevic et al., 1995[4]
O_2	5	86	5.00×10^{-5}	Penkett et al., 1979[5]
O_2 – 0.1% Mn(II)	9	88	8.30×10^{-5}	Martin and Hill, 1987[6]
O_2 – 0.1% Mn(II)	9	80	8.60×10^{-5}	Martin and Hill, 1987[6]
O_2 – 0.01% Mn(II)	9	88	8.30×10^{-6}	Martin and Hill, 1987[6]
O_2 – 0.1% Mn(II)	5	88	5.50×10^{-4}	Martin and Hill, 1987[6]
O_2 – 0.1% Mn(II)	4	88	5.50×10^{-4}	Martin and Hill, 1987[6]
O_2 – 0.1% Mn(II)	4	80	5.70×10^{-3}	Martin and Hill, 1987[6]
O_2 – 0.1% Fe(II)	5	84	3.00×10^{-5}	Martin and Hill, 1987[6]
H_2O_2 (5ppm)	9	82	5.50×10^{-4}	Hoffmann and Calvert, 1985[7]
H_2O_2 (5ppm)	5	82	3.72×10^{2}	Hoffmann and Calvert, 1985[7]
NO_2 (5ppm)	9	86	6.00×10^{1}	Spindler et al., 2003[8]
NO_2 (5ppm)	5	86	1.83×10^{4}	Liu and Abbatt, 2021[9]
O_3 (1ppm)	9	85	1.70×10^{-2}	Hoffmann and Calvert, 1985[7]
O_3 (1ppm)	5	86	4.10×10^{-6}	Hoffmann and Calvert, 1985[7]

Table S2. Values of the in-particle diffusivity.

Oxidant	D ($m^2 s^{-1}$)
O_2	2.1×10^{-9}
H_2O_2	1.4×10^{-9}
NO_2	5.5×10^{-9}
O_3	1.9×10^{-9}
S(IV)	5.00×10^{-10}

Table S3. pH, RH, tipping point radius (*a**), $\gamma_{s,0}$, $\gamma_{rxn,0}$ from CHAI for single droplet S(IV) oxidation studies[10] in a reaction limited regime ($[Y]_0$ = 2.6 M). Since γ_{rxn} is size dependent in the reaction limited regime (leading to a size-independent bulk rate), its value is provided at 20 µm, which is a representative experimental size from Li et al.[10]

Experiment	pH	RH	*a** (µm)	$\gamma_{s,0}$	$\gamma_{rxn,0}$
O_2	9	87	213 (+62/-48)	2.06×10^{-10} ($+2.06\times10^{-10}/-1.95\times10^{-10}$)	1.99×10^{-11}($+5.46\times10^{-12}/-4.42\times10^{-12}$)
O_2	5	86	209 (+46/-38)	9.16×10^{-9} ($+1.72\times10^{-10}/-2.86\times10^{-10}$)	8.44×10^{-10}($+1.94\times10^{-10}/-1.54\times10^{-10}$)
O_2 – 0.1% Mn(II)	9	88	327(+146/-101)	2.63×10^{-9} ($+6.87\times10^{-10}/-2.41\times10^{-10}$)	1.64×10^{-10}($+7.45\times10^{-11}/-4.96\times10^{-11}$)
O_2 – 0.1% Mn(II)	9	80	264 (+82/-63)	6.30×10^{-10} ($+3.26\times10^{-10}/-3.95\times10^{-10}$)	4.62×10^{-11}($+1.44\times10^{-11}/-1.09\times10^{-11}$)
O_2 – 0.01% Mn(II)	9	88	347 (+79/-64)	1.03×10^{-9} ($+6.30\times10^{-11}/-5.27\times10^{-10}$)	5.96×10^{-11}($+1.29\times10^{-11}/-1.09\times10^{-11}$)
O_2 – 0.1% Mn(II)	5	88	158 (+21/-18)	4.81×10^{-8} ($+2.86\times10^{-10}/-5.16\times10^{-10}$)	5.96×10^{-9}($+7.94\times10^{-10}/-6.95\times10^{-10}$)
O_2 – 0.1% Mn(II)	4	88	92.3 (+29.4/-22.1)	5.67×10^{-8} ($+2.86\times10^{-10}/-2.86\times10^{-10}$)	1.24×10^{-8}($+3.87\times10^{-9}/-2.98\times10^{-9}$)
O_2 – 0.1% Mn(II)	4	80	124 (+68/-44)	2.63×10^{-8} ($+6.87\times10^{-10}/-8.59\times10^{-10}$)	4.22×10^{-9}($+2.33\times10^{-9}/-1.49\times10^{-9}$)
O_2 – 0.1% Fe(II)	5	84	218 (+125/-79)	9.74×10^{-9} ($+1.72\times10^{-10}/-8.02\times10^{-10}$)	8.94×10^{-10}($+4.96\times10^{-10}/-3.23\times10^{-10}$)
H_2O_2 (5ppm)	9	82	339 (+125/-91)	2.01×10^{-4} ($+2.00\times10^{-4}/-1.72\times10^{-10}$)	1.16×10^{-5}($+4.41\times10^{-6}/-3.05\times10^{-6}$)
H_2O_2 (5ppm)	5	82	74 (+17/-14)	1.42×10^{-3} ($+4.68\times10^{-4}/-2.36\times10^{-4}$)	3.77×10^{-4}($+8.83\times10^{-5}/-7.22\times10^{-5}$)

Table S4. $\gamma_{rxn,0}$ from CHAI for single droplet S(IV) oxidation studies[10] in a transport limited regime ($[Y]_0$ = 2.6 M).

Experiment	pH	RH	$\gamma_{rxn,0}$
NO_2 (5ppm)	5	86	8.68×10^{-3}($+4.36\times10^{-4}/-6.63\times10^{-4}$)
O_3 (1ppm)	9	85	1.13×10^{-2}($+2.41\times10^{-4}/-2.54\times10^{-4}$)
O_3 (1ppm)	5	85	1.99×10^{-2}($+1.54\times10^{-3}/-1.67\times10^{-3}$)

S3. Supplementary Figures

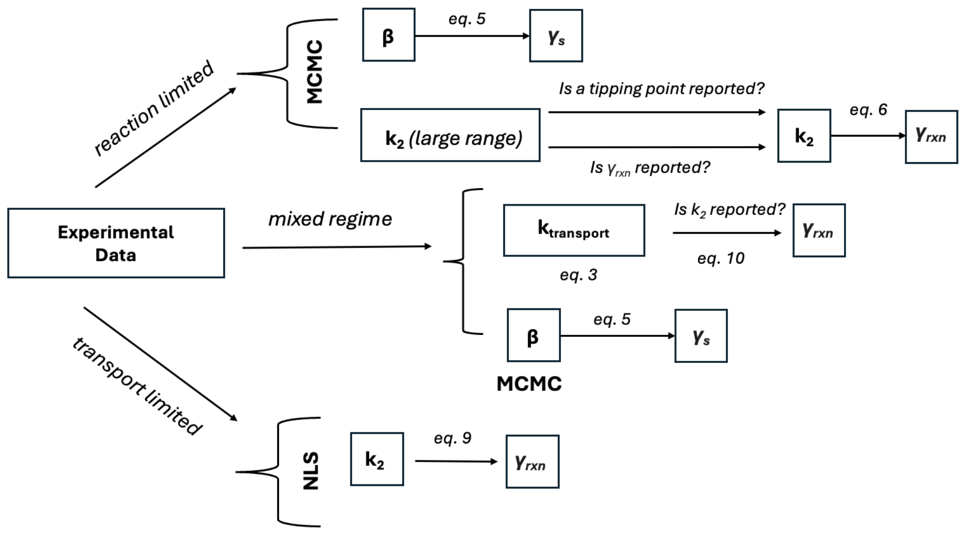

Figure S1. Flowchart explaining how CHAI is used in reaction-limited, transport-limited, and mixed regimes.

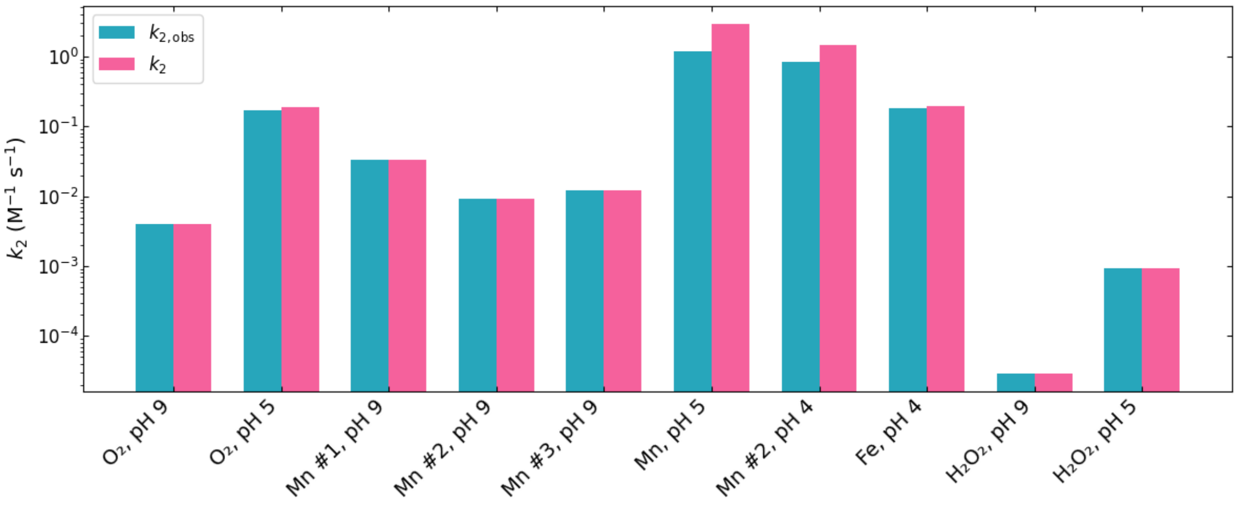

Figure S2. Quantifying the influence of $k_{transport}$ on the observed bulk reaction by equating the bracketed term in eq. 10 with the k_2 values calculated using CHAI in the reaction limited regime ($k_{2,\mathrm{obs}}$).

We note that in one case — O_2 oxidation, 0.1% Mn(II), RH 88 — eq. 10 yields a negative observed rate, which is unphysical. This is likely because our reaction limited derivation for k_2 relies on the "tipping point" reported by Li et al.[10], which differs by 50 µm from the tipping point for the other O_2 + Mn experiment at pH 4, despite similar experimental timescales, and leads to a $k_{2,obs}$ value that is likely an overestimate for the reaction.

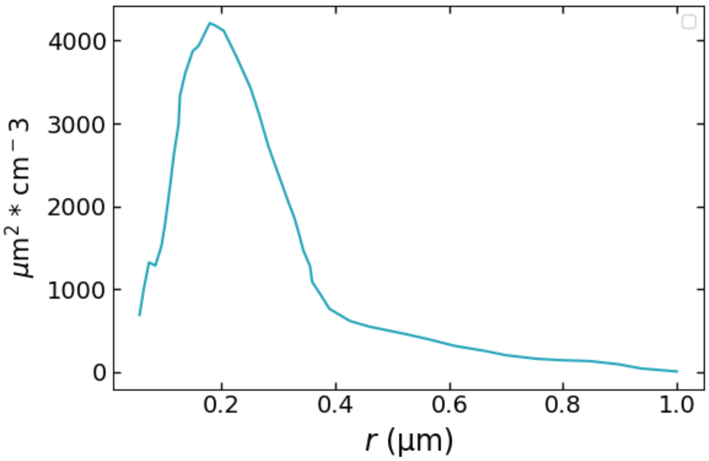

Figure S3. Surface area distribution from Liu et al.[11] used to find the size of interest to compare CHAI and their size-independent reaction for S(IV) oxidation via NO_2.[12]

S4. Additional Multiphase Reactive Systems

Ozonolysis of trans-aconitic acid. Willis and Wilson[2] monitored the loss of particle-phase trans-aconitic acid (AA) via O_3 reaction in a monodisperse population of micrometer sized particles using a quadrupole electrodynamic trap and single droplet mass spectrometer. They found that, in contrast to the NO_2^- ozonolysis, the ozonolysis of AA is dominated by a bulk reaction, with γ_{rxn} coefficients that are an order of magnitude larger than the γ_{s} coefficients. Over the course of the reaction, 88% of reactions between O_3 and AA occur in the bulk solution, despite higher concentrations of adsorbed ozone at the interface, which can be explained by AA's comparatively low surface affinity.

Using CHAI, we fit Willis and Wilson's experimental data to obtain $\beta = 3.50 \times 10^{-7}$ ($-9 \times 10^{-9}/+5 \times 10^{-9}$) m atm^{-1} s^{-1}, which corresponds to $\gamma_{\mathrm{s}} = 3.02 \times 10^{-7}$ ($-8 \times 10^{-9}/+4 \times 10^{-9}$). Using eq. 10 to describe the system (a mixed reaction-transport regime) with a k_2 value ~8×10^{3} M^{-1} s^{-1} (consistent with literature) and $k_{transport}$ = 401 s^{-1} yields $\gamma_{\mathrm{rxn}} = 2.14\times10^{-5}$, which approximately an order of magnitude larger than the reported value of 3.54×10^{-6} for the experiment of interest (a = 9.23 μm, [AA] = 3.2 M, $[O_3]$ = 58.4 ppm). CHAI yields a $\gamma_{\mathrm{overall}}$ of 2.19×10^{-6}, which is comparable to the reported uptake coefficient in Willis and Wilson (4.01×10^{-6}).

Ozonolysis of Fumarate. King et al. used laser Raman tweezers to trap individual droplets containing fumarate, an unsaturated diacid, in an aqueous or organic solvent.[13] They found that the diffusive transport and mass accommodation of O_3 is relatively fast, and that liquid-phase diffusion and reaction are the limiting kinetic steps in this process. Using their model, Wilson et al. found that the ozonolysis of FA occurs mainly at the surface, although the contribution from a parallel bulk reaction is significant ($\gamma_{\mathrm{s}}:\gamma_{\mathrm{obs}}$ is 0.73).[3] Using CHAI, we find that $\beta = 1.08 \times 10^{-3}$ ($-7 \times 10^{-5}/+5.2 \times 10^{-5}$) m atm^{-1} s^{-1}, $\gamma_{\mathrm{s}} = 2.50 \times 10^{-5}$ ($-1.6 \times 10^{-6}/+1.2 \times 10^{-6}$), which is comparable to the value reported by Wilson et al. ($\gamma_{\mathrm{s}} = 1.7 \times 10^{-5}$).

Next, we use the value of k_2 from bulk experiments and compute $k_{transport}$ for the experiment of interest (r = 4.5 μm, [FA] = 0.086 M, $[O_3]$ = 1 ppm) to find γ_{rxn}. We find that for k_2 ~10^{5} M^{-1} s^{-1} and a $k_{transport}$ value of 1688 s^{-1}, $\gamma_{rxn} = 7.31\times10^{-6}$, which is comparable to the bulk uptake coefficient reported by Wilson

et al (6.1×10^{-6}). Both the CHAI-derived scheme and the parameterization by Wilson et al. reflect the fact that this is a surface-dominated process. CHAI yields a $\gamma_{overall}$ = 3.24×10^{-5}, which is comparable to the reported uptake coefficient in Willis and Wilson (2.30×10^{-5}).

S5. Environmental conditions for Table 3

Table S5. Environmental conditions for Beijing winter haze episodes[14,15]used to calculate sulfate production rates in Table 5.

T (K)	277.15
Aerosol pH	4.5
Surface area weighted average particle radius (nm)	218
S_a (cm^2 cm^{-3})	3.7e-5
P_{SO2} (atm)	4.0e-8
P_{HOOH} (atm)	1e-11
P_{NO2} (atm)	6.6e-8
P_{O3} (atm)	1e-9

References

(1) Worsnop, D. R.; Morris, J. W.; Shi, Q.; Davidovits, P.; Kolb, C. E. A Chemical Kinetic Model for Reactive Transformations of Aerosol Particles. *Geophys. Res. Lett.* **2002**, *29* (20), 57-1-57–4. https://doi.org/10.1029/2002GL015542.

(2) Willis, M. D.; Wilson, K. R. Coupled Interfacial and Bulk Kinetics Govern the Timescales of Multiphase Ozonolysis Reactions. *J. Phys. Chem. A* **2022**, *126* (30), 4991–5010. https://doi.org/10.1021/acs.jpca.2c03059.

(3) Wilson, K. R.; Prophet, A. M.; Willis, M. D. A Kinetic Model for Predicting Trace Gas Uptake and Reaction. *J. Phys. Chem. A* **2022**, *126* (40), 7291–7308. https://doi.org/10.1021/acs.jpca.2c03559.

(4) Radojevic, M.; Tyler, B. J.; Hall, S.; Penderghest, N. Air Oxidation of S(IV) in Cloud-Water Samples. *Water. Air. Soil Pollut.* **1995**, *85* (4), 1985–1990. https://doi.org/10.1007/BF01186125.

(5) Penkett, S. A.; Jones, B. M. R.; Brich, K. A.; Eggleton, A. E. J. The Importance of Atmospheric Ozone and Hydrogen Peroxide in Oxidising Sulphur Dioxide in Cloud and Rainwater. *Atmospheric Environ. 1967* **1979**, *13* (1), 123–137. https://doi.org/10.1016/0004-6981(79)90251-8.

(6) Robbin Martin, L.; Hill, M. W. The Effect of Ionic Strength on the Manganese Catalyzed Oxidation of Sulfur(IV). *Atmospheric Environ. 1967* **1987**, *21* (10), 2267–2270. https://doi.org/10.1016/0004-6981(87)90361-1.

(7) Hoffmann, M. R.; Calvert, J. G. *Chemical Transformation Modules for Eulerian Acid Deposition Models. Volume 2. The Aqueous-Phase Chemistry.*; EPA/600/3-85/036; EPA: Research Triangle Park, NC, 1985; p 168. https://ntrl.ntis.gov/NTRL/dashboard/searchResults/titleDetail/PB85198653.xhtml (accessed 2026-03-19).

(8) Spindler, G.; Hesper, J.; Brüggemann, E.; Dubois, R.; Müller, Th.; Herrmann, H. Wet Annular Denuder Measurements of Nitrous Acid: Laboratory Study of the Artefact Reaction of NO2 with S(IV) in Aqueous Solution and Comparison with Field Measurements. *Atmos. Environ.* **2003**, *37* (19), 2643–2662. https://doi.org/10.1016/S1352-2310(03)00209-7.

(9) Liu, T.; Chan, A. W. H.; Abbatt, J. P. D. Multiphase Oxidation of Sulfur Dioxide in Aerosol Particles: Implications for Sulfate Formation in Polluted Environments. *Environ. Sci. Technol.* **2021**, *55* (8), 4227–4242. https://doi.org/10.1021/acs.est.0c06496.

(10) Li, L.-F.; Liu, P.; Huang, Q.; Zhang, X.; Chao, X.; Pang, S.; Wang, W.; Cheng, Y.; Su, H.; Zhang, Y.-H.; Ge, M. Rethinking Urban Haze Formation: Atmospheric Sulfite Conversion Rate Scales with Aerosol Surface Area, Not Volume. *One Earth* **2024**, *7* (6), 1082–1095. https://doi.org/10.1016/j.oneear.2024.05.007.

(11) Liu, T.; Clegg, S. L.; Abbatt, J. P. D. Fast Oxidation of Sulfur Dioxide by Hydrogen Peroxide in Deliquesced Aerosol Particles. *Proc. Natl. Acad. Sci. U. S. A.* **2020**, *117* (3), 1354–1359. https://doi.org/10.1073/pnas.1916401117.

(12) Liu, T.; Abbatt, J. P. D. Oxidation of Sulfur Dioxide by Nitrogen Dioxide Accelerated at the Interface of Deliquesced Aerosol Particles. *Nat. Chem.* **2021**, *13* (12), 1173–1177. https://doi.org/10.1038/s41557-021-00777-0.

(13) King, M. D.; Thompson, K. C.; Ward, A. D.; Pfrang, C.; Hughes, B. R. Oxidation of Biogenic and Water-Soluble Compounds in Aqueous and Organic Aerosol Droplets by Ozone: A Kinetic and Product Analysis Approach Using Laser Raman Tweezers. *Faraday Discuss.* **2008**, *137* (0), 173–192. https://doi.org/10.1039/B702199B.

(14) Cheng, Y.; Zheng, G.; Wei, C.; Mu, Q.; Zheng, B.; Wang, Z.; Gao, M.; Zhang, Q.; He, K.; Carmichael, G.; Pöschl, U.; Su, H. Reactive Nitrogen Chemistry in Aerosol Water as a Source of Sulfate during Haze Events in China. *Sci. Adv.* **2016**, *2* (12), e1601530–e1601530. https://doi.org/10.1126/sciadv.1601530.

(15) Wang, G.; Zhang, R.; Gomez, M. E.; Yang, L.; Levy Zamora, M.; Hu, M.; Lin, Y.; Peng, J.; Guo, S.; Meng, J.; Li, J.; Cheng, C.; Hu, T.; Ren, Y.; Wang, Y.; Gao, J.; Cao, J.; An, Z.; Zhou, W.; Li, G.; Wang, J.; Tian, P.; Marrero-Ortiz, W.; Secrest, J.; Du, Z.; Zheng, J.; Shang, D.; Zeng, L.; Shao, M.; Wang, W.; Huang, Y.; Wang, Y.; Zhu, Y.; Li, Y.; Hu, J.; Pan, B.; Cai, L.; Cheng, Y.; Ji, Y.; Zhang, F.; Rosenfeld, D.; Liss, P. S.; Duce, R. A.; Kolb, C. E.; Molina, M. J. Persistent Sulfate Formation from London Fog to Chinese Haze. *Proc. Natl. Acad. Sci. U. S. A.* **2016**, *113*
